# Prognostic and therapeutic implications of increased ATP citrate lyase expression in human epithelial ovarian cancer

**DOI:** 10.3892/or.2012.1638

**Published:** 2012-01-16

**Authors:** YU WANG, YUNXIA WANG, LIANG SHEN, YINGXIN PANG, ZHEN QIAO, PEISHU LIU

**Affiliations:** Department of Obstetrics and Gynecology, Qilu Hospital, Shandong University, Jinan, Shandong 250012, P.R. China

**Keywords:** ATP citrate lyase, ovarian cancer, prognosis, SREBP-1, RNAi

## Abstract

Altered metabolism is one of the most significant features of cancer cells. ATP citrate lyase (ACL), a key enzyme in *de novo* lipid synthesis, has been reported to be overexpressed or activated in several cancer types. To determine the role of ACL in ovarian cancer progression, we detected ACL expression in human epithelial ovarian cancer tissues. qRT-PCR and western blotting showed higher ACL expression in malignant tissues compared to normal ovarian tissues. Immunohistochemical analysis showed that phosphorylated ACL was increased in ovarian cancer tissues and that its expression correlated well with tumor grade, FIGO stage and poorer prognosis. To explore the therapeutic potential of ACL, we assessed the effect of ACL-siRNA on cellular proliferation and cell cycle distribution. ACL knockdown inhibited cellular proliferation and induced cell cycle arrest in A2780 cells. Taken together, our findings suggest that ACL may contribute to the pathogenesis of human epithelial ovarian cancer, and may serve as a novel therapeutic target.

## Introduction

It has long been recognized that metabolism is altered in human cancers. Warburg first observed enhanced anaerobic glycolysis in cancer in 1926 (1). Elevated glucose catabolism produces excessive levels of the glycolytic end-product, pyruvate. Most of this pyruvate is converted into lactate, but some of it is converted into acetyl-CoA, which, in turn, is used in *de novo* lipid synthesis. Rapidly proliferating cancer cells synthesize fatty acids *de novo* to provide lipids for membrane production and protein modification.

It is increasingly evident that enzymes involved in the lipogenic pathways play direct roles in tumorigenesis and cancer progression (2,3). ATP citrate lyase (ACL), a key enzyme of *de novo* lipid synthesis, is involved in the generation of cytosolic acetyl-CoA and oxaloacetate from citrate and thus contributes to the translocation of acetyl-CoA from mitochondria to cytosol. As a first step in lipid synthesis, pyruvate is converted to acetyl-CoA in the mitochondria. Acetyl-CoA is then incorporated into the TCA cycle which produces citrate in the presence of sufficient amounts of ATP. Accumulated citrate is then exported to the cytoplasm, where it is catalyzed by ACL to generate cytosolic acetyl-CoA, the lipogenic building block (4). The role of ACL in tumor growth has been highlighted by experiments showing that ACL RNAi or the chemical inhibitor SB-204990 suppresses tumor cell proliferation *in vitro*, reduces tumor growth, and induces differentiation *in vivo* (5,6). Migita *et al* reported that ACL was activated via Akt -mediated ACL phosphorylation in lung cancer, while selective ACL inhibition suppressed tumor cell growth both *in vitro* and *in vivo* (7). More recently, investigators have discovered the importance of ACL in promoting tumor invasion via its protection of the glycolytic pathway in glioblastomas (8). However, the role of ACL in human epithelial ovarian cancer has yet to be determined.

Ovarian cancer is the third most common neoplasm of the female reproductive tract and the leading cause of death related to a gynecological malignancy. Epithelial ovarian cancer (EOC) accounts for more than 90% of all ovarian cancers. Several lines of evidence have demonstrated that the dysregulation of lipid metabolism is associated with tumorigenesis and progression of ovarian cancer (9,10). Thus, targeting dysregulated metabolism may be an attractive strategy for ovarian cancer treatment.

In this study, we examined the role of ACL in the pathogenesis of ovarian cancer, and explored its prognostic and therapeutic potential. Overexpression and increased phosphorylation of ACL were found in patients with epithelial ovarian cancer. Phosphorylated ACL was a significant prognostic factor. ACL inhibition by RNAi altered the proliferative behavior and cell cycle distribution of A2780 cells. Our findings suggest that ACL may contribute to ovarian cancer pathogenesis and could serve as a novel therapeutic target.

## Materials and methods

### Cell culture and clinical samples

The human ovarian carcinoma cell line A2780 was obtained from the Type Culture Collection of the Chinese Academy of Sciences (Shanghai, China) and grown in RPMI-1640 supplemented with 10% fetal bovine serum and 1% penicillin/streptomycin in an atmosphere of 5% CO_2_ at 37°C.

Tumor samples were obtained from patients who underwent surgical resection at the Qilu Hospital of Shandong University. All tumors were pathologically diagnosed based on the World Health Organization criteria and staged according to the classification of the International Federation of Gynecology and Obstetrics. Clinicopathological para-meters and patient prognosis data were also obtained. Normal ovaries removed during surgery for benign conditions were collected as controls. This study was approved by the Ethics Committee of Qilu Hospital, and signed informed consent was obtained from each patient.

### Immunohistochemistry and image analysis

Tumor samples were fixed in 10% neutral formalin and embedded in paraffin, and serial 5-μm-thick sections were cut. After deparaffinization, antigen retrieval was performed for 30 min in citrate buffer (pH 6.0). The slides were incubated in blocking serum and then probed overnight at 4°C with primary antibodies against ACL (Abcam, Cambridge, MA), phosphorylated ACL (p-ACL) (Abcam), and SREBP-1 (Santa Cruz Biotechnology, Santa Cruz, CA). Subsequently, biotinylated second antibodies and streptavidin-peroxidase conjugates were applied, and the immunostaining was visualized with 3,3-diaminobenzidine. Finally, the sections were counterstained with hematoxylin. To evaluate the immunoreactivity of ACL and p-ACL, we applied a scoring method that was validated in a previous study (7). The staining intensity of ACL or p-ACL was scored on a scale of 0–4 (0, no staining; 1, weak; 2, moderate; 3, moderate to strong; 4, strong). The patients were classified into two groups: low ACL/p-ACL (scores of 0, 1, or 2) and high ACL/p-ACL (scores of 3 or 4). SREBP-1 expression was also divided into two groups according to the ACL classification.

### Quantitative real-time PCR

Total RNA was extracted from frozen tissue samples using the TRIzol reagent (Invitrogen, Carlsbad, CA) according to the supplier’s protocol. First-strand cDNA was synthesized from total RNA using the ReverTra Ace qPCR RT Kit (Toyobo, Tokyo, Japan). Quantitative RT-PCR was carried out using SYBR-Green Real-Time PCR Master Mix (Toyobo) on a Light Cycler (Roche Applied Science, Indianapolis, IN). The specific primer sequences for ACL and β-actin were as follows: 5′-GAAGGGAGTGA CCATCATCG-3′ (ACL forward); 5′-TTAAAGCACCCAG GCTTGAT-3′ (ACL reverse); 5′-TTGCCGACAGGATGCA GAA-3′ (β-actin forward); and 5′-GCCGATCCACACGG AGTACT-3′ (β-actin reverse). All reactions were performed in at least triplicate. The comparative C_T_ method was used to determine the relative amounts of mRNA (11).

### Cell lysate preparation and western blotting

To obtain total protein lysates, frozen tissue and cells were homogenized in a lysis buffer (Beyotime, Shanghai, China) containing proteinase inhibitors and phosphatase inhibitors. The protein concentration of each lysate was determined using a protein assay reagent kit (Beyotime). Equal amounts of total cellular proteins were separated onto a 10% SDS-PAGE gel and transferred to a PVDF membrane (Millipore, Billerica, MA). The membranes were then incubated with an anti-β-actin (Sigma-Aldrich, St. Louis, MO), anti-ACL (Cell Signaling Technology, Danvers, MA), anti-p-ACL (Cell Signaling Technology), or anti-SREBP-1 (Santa Cruz Biotechnology) primary antibody at 4°C overnight. After being washed and probed with HRP-conjugated secondary antibody, the proteins were detected using ECL Western Blotting Detection Reagents (Millipore).

### RNA interference

Two independent siRNA oligonucleotides for ACL as well as a negative control were purchased from GenePharma (Shanghai, China). The ACL siRNA sequences were designed to correspond to a previous study (5). Transfection was performed using Lipofectamine 2000 (Invitrogen) according to the manufacturer’s protocol. Briefly, 10 μl of a 20 μM siRNA solution and 5 μl of Lipofectamine 2000 were co-incubated in 500 μl of Opti-MEM medium (Invitrogen) for 20 min and overlaid onto cells plated in a 6-well plate.

### MTT assay

After transfection with ACL-siRNA or negative control siRNA for the indicated time periods, cells were incubated with 5 mg/ml MTT (Sigma-Aldrich) for 4 h and then mixed with DMSO after the supernatant was removed. Cell viability was quantitated according to the dye absorption (A) at 550 nm (A1) and 630 nm (A2) with an automatic multiwall spectrophotometer (Bio-Rad Laboratories, Richmond, CA). Each experiment was repeated in triplicate.

### Flow cytometry assay

For cell cycle analysis, cells were harvested and fixed with 70% ice-cold ethanol at 4°C overnight. After washing with PBS, the cells were suspended with 200 μg RNaseA (1 mg/ml) at 37°C for 30 min and later stained with 800 μl (100 μg/ml) propidium iodide (Invitrogen) at 37°C for 30 min. For the apoptosis analysis, cells were washed, resuspended in ice-cold binding buffer, and subsequently incubated with 5 μl Annexin-V-FITC (Jingmei Biotech, Shanghai, China) and 10 μl propidium iodide for 15 min. Cell cycle and cell apoptosis analyses were performed with a flow cytometer (FCM) (BD Biosciences, San Jose, CA). Each experiment was repeated in triplicate.

### Statistical analysis

The data from the cell culture experiments were compared using Student’s t-tests. For the clinical samples, the correlations between ACL/p-ACL expression and clinicopathological parameters were assessed using Fisher’s exact test. Univariate survival analysis was performed using the Kaplan-Meier method and log-rank statistics for comparisons of survival curves. Multivariate survival analysis was determined by the Cox regression model. Comparisons of ACL expression between ovarian cancer tissues and normal tissues were carried out using the Mann-Whitney U Test. For all analyses, a p-value of <0.05 was considered statistically significant. Statistical analyses were performed using SPSS software.

## Results

### ACL is overexpressed in and serves as a prognostic factor for human epithelial ovarian cancer

**Figure 1:**
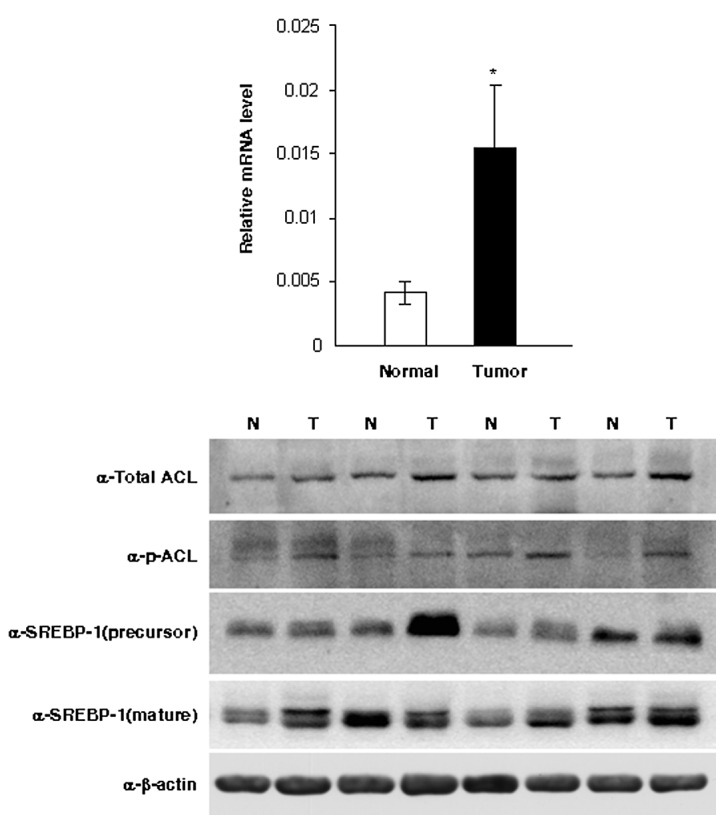
ACL expression in epithelial ovarian cancer tissues. (A) Quantitative real-time PCR analysis of ACL in human epithelial ovarian cancer tissues and normal ovarian tissues. The level of ACL mRNA was normalized to the level of human β-actin expression. The ACL mRNA level was increased in epithelial ovarian cancer tissues compared to normal tissues. Data are expressed as mean ± SEM. ^*^p<0.05. (B) ACL, p-ACL and SREBP-1 expression detected by western blot analysis. Representative western blot assays from four pairs of frozen samples of ovarian tumors and normal tissues are shown.

To evaluate the role of ACL in epithelial ovarian cancer, we assessed ACL expression in 18 epithelial ovarian cancer tissues and 12 normal ovarian tissues. ACL mRNA was upregulated 3.7-fold in ovarian cancer tissues compared to normal tissues (p<0.05; Fig. 1A). Western blot analysis confirmed the increased expression of ACL and p-ACL protein in ovarian cancer tissues compared to normal tissues (Fig. 1B).

SREBP-1 is a basic-helix-loop-helix-leucine zipper protein that regulates the transcription of lipogenic enzymes. To dissect the relationship of SREBP-1 with ACL in ovarian cancer, we detected SREBP-1 expression by western blot analysis (Fig. 1B). However, our data showed that ACL and p-ACL expression were independent of either the 125-kDa precursor form of SREBP-1 or the 68-kDa mature and cleaved nuclear form (Fig. 1B).

**Figure 2:**
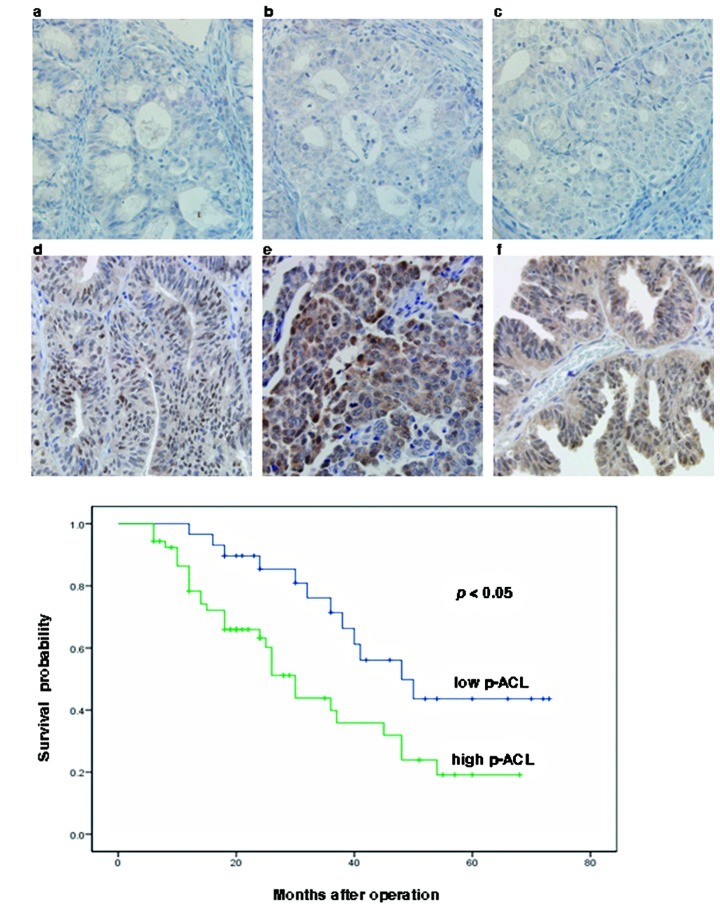
Immunohistochemical analyses of ACL, p-ACL and SREBP-1 in epithelial ovarian cancer tissues. (A) Representative images of low and high ACL (a and d), p-ACL (b and e), and SREBP-1 (c and f) immunostaining (magnification, ×400). (B) Overall survival of ovarian cancer patients with reference to p-ACL expression. Differences between the two groups were evaluated with the log-rank test.

To determine whether ACL was associated with clinicopathologic parameters, ACL and p-ACL expression in the 82 epithelial ovarian cancer samples were analyzed by immunohistochemistry. The clinicopathological characteristics of these 82 patients are summarized in Table I. Immunohistochemical results showed that ACL and p-ACL were expressed at different levels in different samples, which were classified as low expression (scores of 0, 1, or 2) or high expression (scores of 3 or 4) groups (Fig. 2A). High ACL and high p-ACL scores were recorded in 51 (62%) and 53 (65%) cases, respectively. Comparisons of p-ACL levels and clinicopathological para-meters revealed that high p-ACL expression was significantly correlated with poor differentiation (p=0.044) and advanced FIGO stage (p=0.020; Table I). Total ACL expression was not correlated with any of the clinicopathological parameters studied (Table I).

We also detected SREBP-1 expression using immunohistochemistry (Fig. 2A). Statistical analysis showed that neither ACL nor p-ACL immunostaining patterns were correlated with SREBP-1 expression (Table I). These findings were consistent with our western blot analyses. In our study, ACL and p-ACL expression was detected in both the cytoplasm and the nucleus of tumor cells in some samples.

Next, we examined the relationship between p-ACL expression and survival in patients with epithelial ovarian cancer (n=82). The median survival for patients with high p-ACL ovarian cancer was significantly shorter than the median survival for those patients with low p-ACL ovarian cancer (30.0 months; 95% CI, 25.0–34.9 months vs. 48.0 months; 95% CI, 32.1–63.9 months; p=0.010), and the 5-year survival rates in these two groups were 19.1% and 43.6% (p=0.010; Fig. 2B), respectively. Univariate survival analysis showed that age, tumor grade, FIGO stage and p-ACL expression were significantly associated with survival (p<0.05). Furthermore, in a multivariate Cox regression model with these significant covariates, age and FIGO stage were significant factors for prediction of a poor prognosis (p=0.001 and p=0.031, respectively). Total ACL expression was not significantly associated with survival (data not shown).

### Knockdown of ACL by siRNA impairs the proliferation of A2780 cells

**Figure 3:**
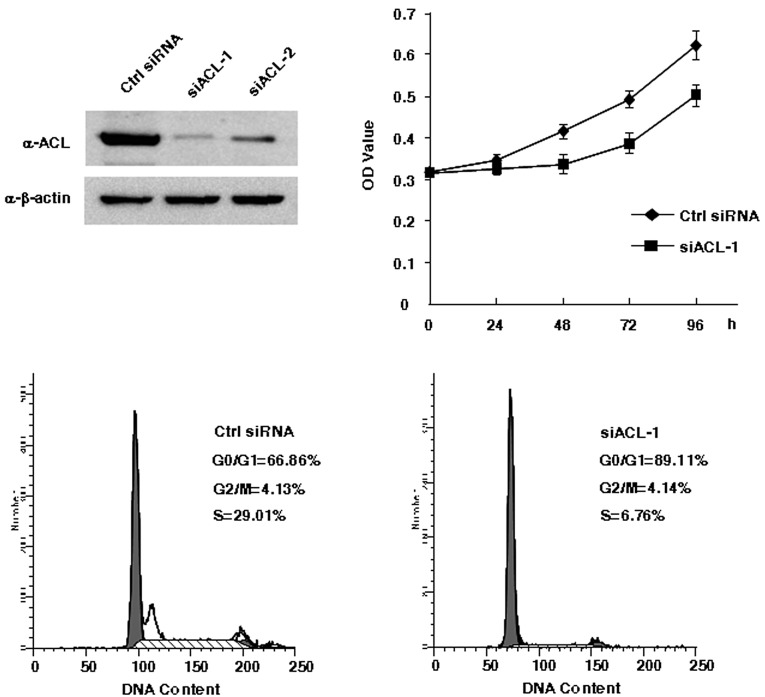
Effects of ACL knockdown on cellular proliferation and cell cycle progression in A2780 cells. (A) ACL protein levels in A2780 cells transfected with negative control siRNA or two different ACL siRNA oligonucleotides were determined by western blot analysis 72 h after transfection. (B) MTT assay was performed to examine cellular proliferation after transfection. Cellular proliferation was measured every 24 h and significant differences were observed between the siACL-1-treated cells and control siRNA-treated cells at 48, 72 and 96 h (p<0.05). Data are expressed as mean ± SD from a representative experiment. (C) Cell cycle phase distribution was detected by flow cytometry 72 h after siRNA transfection. ACL knockdown blocked cell entry into the S-phase. The percentages of cells in the G1, G2/M, and S phases are indicated. The figure shown is representative of triplicate experiments.

To study whether ACL has potential as a therapeutic target in ovarian cancer, we used ACL-specific siRNAs to selectively reduce ACL gene expression in A2780 cells. Both oligonucleotides (siACL-1 and siACL-2) efficiently reduced total ACL protein levels. However, because western blot analyses showed that siACL-1 was more effective (Fig. 3A), siACL-1 was used in all following experiments. MTT assays were performed to examine the effect of ACL inhibition on cellular proliferation. After transfection with siRNA, the ACL knockdown cells proliferated more slowly than did the control cells, and significant differences between the ACL knockdown cells and control cells were observed at 48, 72 and 96 h (p<0.05, Fig. 3B).

### Effect of ACL-siRNA on cell cycle progression and apoptosis

After transfection with siRNAs for 72 h, cell cycle distribution and apoptosis were analyzed with flow cytometry. The cell cycle analysis indicated that ACL knockdown induced a progressive reduction in the S-phase population that was associated with a progressive parallel accumulation of G1-phase cells (negative control siRNA, G1, 66.86%; S, 29.01%; and G2-M, 4.13%; siACL-1, G1, 89.11%; S, 6.76%; and G2-M, 4.14%; Fig. 3C). We concluded that cell cycle arrest may contribute to the cellular proliferation defect in the ACL-siRNA-treated cells. We did not observe significant change in apoptosis after ACL knockdown (data not shown).

## Discussion

Altered metabolism is one of the most significant features of cancer cells. High glycolysis and lipogenesis occur in cancers to satisfy the increased demand for bioenergy and macromolecules need for autonomous growth. It has been reported that *de novo* fatty acid synthesis occurs at low rates in normal tissues because normal cells acquire lipids via circulation; in contrast, fatty acid synthesis occurs at very high rates in tumor tissues (12). Ookhtens *et al* confirmed that almost all fatty acids are derived from *de novo* synthesis in tumor cells despite an abundant supply of extracellular lipids (13). Thus, glucose and lipid metabolism-targeting therapeutics have attracted great attention (14,15).

Accumulating evidence indicates that various steps of lipid synthesis contribute to cancer progression. Akt was originally identified as a potent proto-oncogene, and constitutive PI3K/Akt activation is commonly observed in a variety of cell types (16,17). PI3K/Akt signaling is sufficient to stimulate glucose uptake, as well as glycolysis and fatty acid biosynthesis in cancer cells (18). The blockade of HMG CoA reductase is widely considered to be a useful strategy for inhibiting cancer cell growth and inducing apoptosis both *in vitro* and *in vivo* (19,20). Fatty acid synthase (FASN), the enzyme responsible for the terminal steps in *de novo* fatty acid synthesis, is highly expressed in a wide variety of human epithelial cancers (21,22). Furthermore, lipogenic enzymes that function upstream of FASN such as acetyl-CoA carboxylase-a (ACACA) and ATP citrate lyase (ACL) are also elevated in various cancers. These enzymes have also been considered to play critical roles in tumorigenesis and cancer progression (23,24).

ACL regulates a key step that can convert high glycolytic flux into increased lipid synthesis. ACL overexpression or activation has been reported in bladder, breast, liver, stomach and lung tumors (7,25–28). As the first committed step in the glucose-dependent lipid synthesis pathway, ACL inhibition can affect the cholesterol, isoprenoid, and fatty acid synthesis pathways in combination. Thus, ACL could be a particularly attractive target for ovarian cancer therapy.

We detected ACL expression in human epithelial ovarian cancer and normal ovarian tissues using quantitative RT-PCR and western blot analysis. Significantly elevated levels of ACL and p-ACL were observed in ovarian cancer tissues compared to normal tissues, which suggests that ACL could promote ovarian cancer tumorigenesis and progression.

The prognoses of women with epithelial ovarian cancers are invariably poor. Recent attention has focused on the identification of potential biological prognostic markers. We assessed ACL expression in epithelial ovarian cancer tissues using immunohistochemistry and analyzed the relationships between ACL expression and the clinicopathological characteristics of the patients. Our findings showed that p-ACL expression was significantly correlated with tumor grade and FIGO stage, suggesting that increased ACL phosphorylation could be generally related to the aggressive behavior of ovarian cancer. p-ACL expression was significantly correlated with overall survival. Thus, ACL may be a useful marker for epithelial ovarian cancers with especially poor prognoses.

As the rate-limiting enzyme that is responsible for generating cytosolic acetyl-CoA from citrate, ACL is considered to be localized in the cytoplasm. However, we observed both nuclear and cytoplasmic localization of ACL and p-ACL proteins. This finding is consistent with a previous study in lung tissue (7). To date, the function of ACL in the nucleus remains unclear, and further research is required to clarify the role of differential ACL cellular localization.

SREBP-1 is a transcription factor that is involved in cholesterol and lipid metabolism, and it has been reported that ACL is transcriptionally regulated by SREBP-1 (29,30). We assessed SREBP-1 protein levels in epithelial ovarian cancer tissues using western blot analysis and immunohistochemistry. The results showed that both ACL and p-ACL expression were independent of SREBP-1.

Given the positive role of ACL in cancer pathogenesis, we sought to determine whether the decreased expression of ACL influenced the biological behavior of A2780 cells. We observed markedly impaired proliferation coupled with delayed S-phase entry in cells transfected with ACL-siRNA.

In conclusion, our findings indicate that ACL is overexpressed in and could serve as a prognostic factor for human epithelial ovarian cancer. ACL knockdown inhibits cellular proliferation and induces cell cycle arrest *in vitro*. The present study suggests that ACL may be a novel therapeutic target for epithelial ovarian cancer. However, more ovarian cancer cell lines should be investigated, and further research will be required to elucidate the detailed changes in lipid metabolism induced by ACL inhibition in ovarian cancer cells.

## Figures and Tables

**Table I:** Relationships between ACL/p-ACL expression and clinicopathological parameters in epithelial ovarian cancer.

	N	ACL low, n	ACL high, n	p-value	p-ACL low, n	p-ACL high, n	p-value
Age (years)	82	31	51	0.649	29	53	0.164
<60	44	18	26		19	25	
≥60	38	13	25		10	28	
Tumor grade	82	31	51	0.623	29	53	0.044
1	10	5	5		7	3	
2	19	6	13		7	12	
3	53	20	33		15	38	
Preoperative maximal diameter of tumor (cm)	82	31	51	0.365	29	53	0.760
<10	63	22	41		22	41	
≥10	14	7	7		6	8	
Unknown	5	2	3		1	4	
FIGO stage	82	31	51	0.610	29	53	0.020
I, II	21	9	12		12	9	
III, IV	61	22	39		17	44	
Histological type	82	31	51	1.000	29	53	0.788
Serous	62	24	38		21	41	
Others	20	7	13		8	12	
SREBP1	82	31	51	0.113	29	53	0.248
Low	36	10	26		10	26	
High	46	21	25		19	27	
